# A Quantum Hybrid PSO Combined with Fuzzy *k*-NN Approach to Feature Selection and Cell Classification in Cervical Cancer Detection

**DOI:** 10.3390/s17122935

**Published:** 2017-12-19

**Authors:** Abdullah M. Iliyasu, Chastine Fatichah

**Affiliations:** 1 Electrical Engineering Department, College of Engineering, Prince Sattam Bin Abdulaziz University, Al-Kharj 11942, Saudi Arabia; 2 School of Computing, Tokyo Institute of Technology, Yokohama 226-8502, Japan; 3 School of Computer Science and Technology, Changchun University of Science and Technology, Changchun 130022, China; 4 Informatics Department, Institut Nepuluh Nopember, ITS Campus, Surabaya 60111, Indonesia; chastine@cs.its.ac.id

**Keywords:** computational intelligence, quantum hybrid intelligent systems, quantum machine learning, medical image processing, disease diagnosis, Fuzzy *k*-NN, quantum-behaved PSO, cervical smear images, cancer detection

## Abstract

A quantum hybrid (QH) intelligent approach that blends the adaptive search capability of the quantum-behaved particle swarm optimisation (QPSO) method with the intuitionistic rationality of traditional fuzzy *k*-nearest neighbours (Fuzzy *k*-NN) algorithm (known simply as the Q-Fuzzy approach) is proposed for efficient feature selection and classification of cells in cervical smeared (CS) images. From an initial multitude of 17 features describing the geometry, colour, and texture of the CS images, the QPSO stage of our proposed technique is used to select the best subset features (i.e., global best particles) that represent a pruned down collection of seven features. Using a dataset of almost 1000 images, performance evaluation of our proposed Q-Fuzzy approach assesses the impact of our feature selection on classification accuracy by way of three experimental scenarios that are compared alongside two other approaches: the All-features (i.e., classification without prior feature selection) and another hybrid technique combining the standard PSO algorithm with the Fuzzy *k*-NN technique (P-Fuzzy approach). In the first and second scenarios, we further divided the assessment criteria in terms of classification accuracy based on the choice of best features and those in terms of the different categories of the cervical cells. In the third scenario, we introduced new QH hybrid techniques, i.e., QPSO combined with other supervised learning methods, and compared the classification accuracy alongside our proposed Q-Fuzzy approach. Furthermore, we employed statistical approaches to establish qualitative agreement with regards to the feature selection in the experimental scenarios 1 and 3. The synergy between the QPSO and Fuzzy *k*-NN in the proposed Q-Fuzzy approach improves classification accuracy as manifest in the reduction in number cell features, which is crucial for effective cervical cancer detection and diagnosis.

## 1. Introduction

Hybrid intelligent systems (HIS) simultaneously integrate (or combine) two or more intelligent approaches, such as fuzzy techniques, genetic algorithms, neural networks, agent-based techniques, case-based reasoning, and other computationally (or artificially) intelligent approaches conducive to overcome individual limitations and achieve synergetic outcomes. Such hybridisation offers the capability to handle real-world complex problems involving imprecision, uncertainty, vagueness, high-dimensionality, etc. [1]. HIS systems are applied in almost every area of life, but notable applications can be found in science, technology, business, commerce, and medicine.

Cervical cancer is one of the most common lethal malignant diseases among women. Thankfully, however, with improvements in medical technologies, over the past few years, it is easier to detect such disease at an early stage by doing Pap smear image tests. These tests typically involve filtering out abnormal cervical cells [2] and use of the results to detect precancerous changes in cervical cells based on colour and shape properties of their nuclei and cytoplasm [3]. Screening for cervical cancer by Pap smear image tests provide an inference regarding the presence of the Papilloma virus that is responsible for cervical cancer [4]. However, performing the Pap smear test manually is known to be a time-consuming and error-prone exercise that is further exacerbated by the lack of adequate pathology expertise [5]. In addition, due to subjective disparity from different cytologists, the results of the screening often show a lot of inconsistencies [6], thereby further compromising the screening process [7]. Moreover, since hundreds of images need to be analysed daily, the manual screening process to classify the cells is a challenging pursuit that is susceptible to error [8]. A single cell from the Pap smear test can be classified into one of seven classes [9,10,11,12], which are superficial squamous, intermediate squamous, columnar, mild dysplasia, moderate dysplasia, severe dysplasia, and carcinoma in situ.

Meanwhile, in furtherance of improving the accuracy of automated cervical cell detection systems, dynamic segmentation techniques are required to delineate the contours of the cytoplasm and nucleus in the cell images from Pap smear tests. This has led to the proposal of numerous approaches aimed at improving the assessment of Pap smear image test results. 

Some studies related to the cervical cancer detection classification of various cervical cell features are presented in [8,9,10,11,12,13,14]. The study in [8] uses 14 features and five classifiers to validate their classification results with focus on digital imaging colposcopy. In [11], a neuro-fuzzy method was used to classify the 20 features in cervical cell types; while in [13] an automatic cervical cell segmentation and classification method was applied on three datasets of Pap smeared images. Therein, nine features were used and the results obtained were compared alongside five other classification methods. A support vector machine technique based on recursive feature elimination (SVM-RFE) was used to select the features and classify the cervical cell types in [14]. In that study, eleven (11) nuclei features and nine cytoplasm features were used to differentiate the cervical cell types. The study in [14] combined four feature selection approaches with the traditional support vector machine (SVM) algorithm in order to classify the cell types. It was reported therein that the accuracy of the classification results depended on good choice of features, which further indicates the importance of choosing the best subset of features enhances to accuracy of classification results.

In terms of feature selection, three methods: filter, wrapper, and hybrid methods, are widely used in the literature [15]. The filter method is considered computationally fast, easy to interpret, and is scalable for high-dimensional data [16]. Nonetheless, equipped with advanced machine learning algorithms to select the best feature subsets, the wrapper and hybrid methods are known to demonstrate better performances than the filter methods. Besides, hybrid methods commonly use supervised learning techniques and swarm-based intelligent methods as integral components of their feature selection. Many previous studies utilised the swarm intelligence algorithms for their feature selection stages, among which is the particle swarm optimisation (PSO) that is widely used to solve optimisation problems [17]. 

Recently, quantum machine learning, which is an approach integrating quantum mechanics into traditional machine learning approaches, has been used to further understand and enhance the learning process. One of such techniques is the quantum-behaved particle swarm optimisation (QPSO), which is a variant of the standard PSO algorithm, that was proposed in [18] by exploiting some proven properties of quantum mechanics. Among other superlative properties, QPSO eliminates the velocity term and control parameters that are used in the traditional PSO approach [17]. This ensures that, in comparison with the original PSO algorithm [18], the QPSO offers improved performance in terms of its search capability. 

Since that effort, numerous approaches have been suggested to further enhance the QPSO. Among them, a new QPSO (NQPSO) algorithm was proposed in [19] to further improve on the QPSO by employing and balancing the choice of local and one global neighbourhood search strategies. Similarly, in [20] an improved QPSO (IQPSO) algorithm was proposed and utilised for visual features selection (VFS). Overall, the standard QPSO approach was proposed to deal premature convergence, and it is simple and easy to understand [19]. The use of a global optimal to determine the best subset features makes the QPSO a veritable choice to accelerate convergence in feature selection tasks.

Therefore, the study presented in this work exploits the proven versatility of the QPSO algorithm as the main component of a new hybrid approach for selecting features of cervical cells in Pap smeared images by blending the QPSO with the intuitionistic descriptiveness of Fuzzy *k*-Nearest Neighbours (Fuzzy *k*-NN) algorithms. Specifically, the best choice of subset features is enhanced by combining the QPSO with the Fuzzy *k*-NN, which itself is an extension of the *k*-nearest neighbours (*k*-NN) algorithms but with fuzzy intuition integrated into it [21,22,23]. As envisaged, this hybridisation leads to modest improvements in cervical cell classification accuracy.

To validate the expected potency of the proposed technique, the Herlev dataset [24], which contains original and segmented images collated using the CHAMP software [10,11,12], is used. To establish the cogency of the outcomes from our proposed technique, we utilised 17 geometric, chromatic, and textural features that are employed in some available literature [3,11,12] to describe our cervical cell images. The acuteness of the QPSO unit of the proposed technique ensures that the best features are selected and then pruned down to a collection of seven features. This feature selection step, combined with the rationality from the Fuzzy *k*-NN guarantees improvement in cell classification accuracy, which is crucial in cervical cancer prediction.

The remainder of the paper is organised as follows: advances in cervical cell classification for smeared images are highlighted in the next section as well as a succinct overview of the QPSO and Fuzzy *k*-NN algorithms including arguments supporting their adoption as the core units of our proposed approach to feature selection and cell classification for cervical cancer detection. These two units and how they coalesce into our quantum hybrid (QH) system are discussed in Section 3, while experimental results to demonstrate the utility of our proposed technique are reported in Section 4.

## 2. Overview of Cell Classification in Smeared Cervical Images, Quantum-Behaved PSO and Fuzzy *k*-NN

As mentioned earlier, in this section we will highlight the advances made in cell classification for smeared cervical images. Additionally, we will present a succinct overview of the QPSO and Fuzzy *k*-NN algorithms and conclude with a few arguments supporting their adoption as the core units of our proposed approach to feature selection and cell classification in cervical cancer detection.

### 2.1. Cell Classification in Smeared Cervical Images

Cervical cancer is a malignant cancer that forms in cervical tissues (i.e., the organ that connects the uterus to the vagina) [14]. The Pap smear image test is one of the first procedures used to extract medical inference regarding the presence of the Papilloma virus, which is known to be responsible for causing cervical cancer [3]. Pap smear image tests also provide a window for early detection and treatment before the condition deteriorates [6]. A single-cell pap smear image can be classified into one of the seven classes presented in Table 1 [9,10,11,12].

As seen from the table, abnormal cervical cells that have undergone precancerous changes (called a dysplastic cell) are further divided into four main phases. The first phase, called mild dysplasia, occurs when the nucleus grows larger and brighter than normal. In the second phase, called moderate dysplasia, the nucleus becomes darker and larger in size. The third phase, known as severe dysplasia, occurs when the size and texture of both the nucleus and cytoplasm change: the nucleus grows (in size) while becoming darker with strange shapes, and the cytoplasm also becomes darker but smaller in size. The last phase, known as Carcinoma in situ, is characterised by a very large nucleus and it occurs at a point when cytology strongly suggests malignancy.

**Figure 1:**
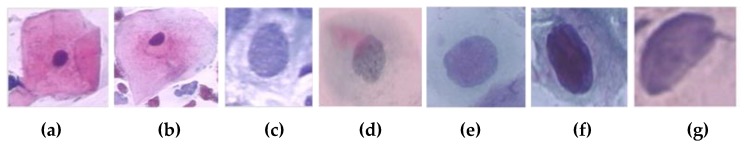
A single cell cervical smear image: (**a**) superficial squamous, (**b**) intermediate squamous, (**c**) columnar, (**d**) mild dysplasia, (**e**) moderate dysplasia, (**f**) severe dysplasia, (**g**) carcinoma in situ [2,9,10,11,12,13].

Knowledge of these cell attributes allows us to describe cells in cancerous and precancerous stages since they are marked by many changes in morphology and architecture, including geometry (in terms of both shape and size) of the cytoplasm and nucleus, changes in nuclear-cytoplasm ratios, and others [15,16,17]. Some sample results for Pap smear image tests are shown in Figure 1.

### 2.2. Quantum-Behaved Particle Swarm Optimisation (QPSO)

Quantum computing is a new and exciting computing paradigm that is tailored towards exploiting the physical attributes of quantum mechanics to harness information processing [25]. Many new applications have been proposed either for use with quantum computing hardware or just to exploit some of the confounding properties of quantum mechanics to enhance traditional computing protocols, notably those in the areas of image processing, quantum machine learning (QML), and general areas of computational intelligence [25,26,27,28]. 

QML, which incorporates integration of ‘quantumness’ into traditional machine learning algorithms, and its application in different domains is an emerging sub-discipline that seems to receiving increased attention [29]. Among others, a major objective of this study is to explore the integration of QML techniques (specifically, QPSO) in image-based cervical cancer detection [29,30].

We begin our discussion of the QPSO with a pithy highlight of the standard particle swarm optimisation (PSO) algorithm. PSO was proposed (by Eberheart and Kennedy in 1995 [21]) to mimic the social behaviour of a swarm of birds searching for food in a predefined space, with each of them behaving in accordance with the expected intelligence of the swarm population in such an environment [20]. Furthermore, the scenario imposes the additional restriction that there is only one source of food and none of the birds has prior knowledge of this location. Although rather inefficacious, based on the foregoing scenario, the easiest solution would be to trail any bird that perchance stumbles into this location. Consequently, all the birds in the population would traverse (albeit, randomly) the same path to the food source irrespective of their own proximity to the source and the remaining birds in the population. In PSO parlance, each single solution in the search space is called a particle, like a bird. PSO is initialised with random particles (solutions) and an optimal solution is determined within the search space by updating each generation. All particles have fitness values and velocities with which they fly over the search space as they follow paths covered by particles that are perceived as better solutions [19].

The *d* dimension of the *i*th particle is represented as *X_i_* = (*x_i_*_1_, *x*_*i*2_, …, *x*_*i*d_) and for each generation each particle is updated using two ‘best’ values [16]. The first of these solutions, called *P_i_best_* (personal best), is the best solution for a specific particle, while the second one, called *P_global_* (global best) is the best value of any particle in the population. The fitness function of particle *X* (denoted as *F*(*X*)), is defined using the F1 score (which will be presented much later in Equation (29)).

The quantum-behaved version of the PSO (i.e., the QPSO) was proposed to improve on the capabilities of the PSO algorithm. In it, the probability of the particle appearing in position *X* may be obtained from the quantum mechanical interpretation of the wave function of the particle at current position (*t*) as described in Equation (1) [20].

(1) $$\psi \left(x\left(t\right)\right)=\frac{1}{\sqrt{Q}}e^{-\frac{\left|m_{best}\left(t\right)-X\left(t\right)\right|}{Q}}$$

where the parameter *Q* depends on the mean of best and current positions of the particles and it helps to specify the search scope for a particle; and *m_best_* is calculated as the mean of the best positions of all particles (*S*) in the population, such that:

(2) $$m_{best}=\frac{1}{S}\sum P_{i\_best}$$

The parameter *Q* and the position *X* are updated according to the constraints in Equations (3)–(5).

(3) $$Q\left(t\right)=2\cdot \propto \cdot |m_{best}\left(t\right)-X\left(t\right)|,$$

(4) $$X\left(t+1\right)=p\left(t\right)-\propto \cdot |m_{best}\left(t\right)-X\left(t\right)|\cdot ln\frac{1}{u\left(t\right)},\;\mathrm{if}\;s\left(t\right)\geq 0.5$$

and

(5) $$X\left(t+1\right)=p\left(t\right)-\propto \cdot |m_{best}\left(t\right)-X\left(t\right)|\cdot ln\frac{1}{u\left(t\right)},\;\mathrm{if}\;s\left(t\right)<0.5,$$

where *u* and *s* are uniformly distributed random numbers in the interval (0,1), the parameter *α* is the contraction-expansion coefficient [18], and *p*(*t*) takes the form defined in Equation (6).

(6) $$p\left(t\right)=\phi \left(t\right)\cdot P_{i\_best}\left(t\right)+\left(1-\phi \left(t\right)\right)\cdot P_{global}\left(t\right),$$

where *ϕ* is a uniformly distributed random number in the interval (0,1), and to update the new best position of particle *I* (*P_i_best_* (*t* + 1)), Equation (7) is used.

(7) $$P_{i\_best}\left(t+1\right)=\{\begin{array}{c} X\left(t\right),\;if\;F\left(X\left(t\right)\right)>F\left(P_{i\_best}\left(t\right)\right)\\ P_{i\_best}\left(t\right),\;if\;F\left(X\left(t\right)\right)\leq F\left(P_{i\_best}\left(t\right)\right) \end{array}$$

The Pseudocode in Table 2 outlines the steps to execute the QPSO algorithm (as discussed in [18,19]).

**Figure 2:**
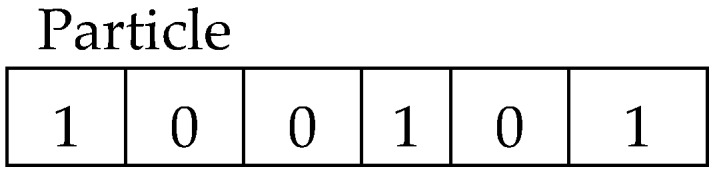
Illustration of particle encoded as a binary string where the bit value ‘1’ denotes a selected feature and ’0’ denotes a non-selected feature.

As outlined in the pseudocode, each particle is encoded into a binary string whose length is equal to the size of feature vector. A value of ‘1’ indicates that a feature is selected, whereas a value of ‘0’ indicates otherwise. In this manner, each particle is encoded into a binary string as presented in Equation (8) and further depicted in Figure 2 [19].

(8) $$x=\{\begin{array}{c} 1,\;\mathrm{if}\;Sigmoid\left(x\right)>U\left(0,1\right)\\ 0,\;\mathrm{otherwise} \end{array},$$

where *Sigmoid*(*x*) = 1/(1 + *e*^−x^) and *U*(0,1) is a uniformly distributed random number in the interval (0,1).

The 100101 six-bit string depicting a particle (shown in Figure 2), shows that the first, fourth, and sixth features are selected, whereas the second, third, and fifth features are not selected. The fitness value of each particle is calculated based on the accuracy of the classification results.

### 2.3. Fuzzy k-Nearest Neighbours (Fuzzy k-NN)

Fuzzy *k*-nearest neighbours (Fuzzy *k*-NN) is an extension of the standard *k*-nearest neighbours (*k*-NN) algorithm but with fuzzy intuition is integrated into it [22]. More precisely, fuzzy theory is used to generalise definitions of the *k*-NN membership values of the data in each class as defined in Equation (9).

(9) $$u\left(x,c_{i}\right)=\frac{\sum_{k=1}^{K}u\left(x_{k},c_{i}\right).d{\left(x,x_{k}\right)}^{\frac{-2}{m-1}}}{\sum_{k=1}^{K}d{\left(x,x_{k}\right)}^{\frac{-2}{m-1}}},$$

where *u*(*x*, $c_{i}$) is membership values of data *x* in the class $c_{i}$, *k* value is the number of nearest neighbours, *u*(*x_k_*, $c_{i}$) is membership value of *k* nearest neighbours’ data *x* in the class $c_{i}$, *d*(*x*, $x_{k}$) is distance between data *x* and *k* nearest neighbours, and *m* is weight exponent, which should be greater than 1.

The pseudocode in Table 3 outlines the Fuzzy *k*-NN algorithm (as discussed in [22,23]).

## 3. Methodology for Quantum Hybrid Approach to Cervical Cancer Feature Selection and Classification

Pap cervical smear (CS) images are rich in various features like colour, shape, and texture. The process of accurate extraction of these unique visual features from the images is very crucial in developing an automated cancer screening process [8].

**Figure 3:**
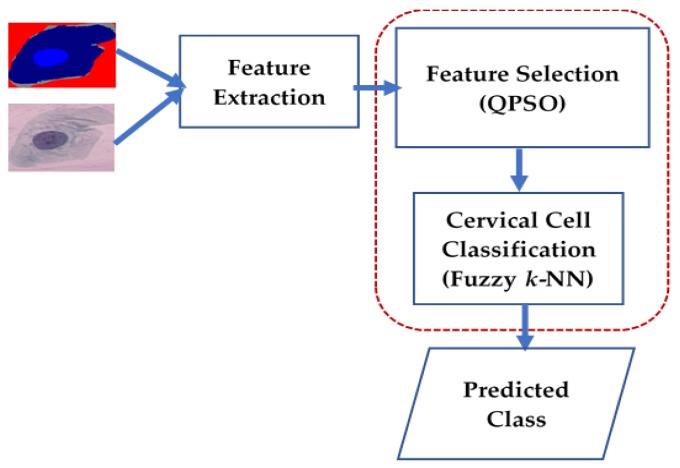
Layout of proposed quantum hybrid (Q-Fuzzy) technique to select and classify cells in smeared cervical images.

The layout of our proposed quantum hybrid technique (QHT) that combines the quantum-behaved PSO with the Fuzzy *k*-NN (henceforth referred to as the Q-Fuzzy approach) to select appropriate features for accurate classification of cervical cells in smeared images is presented in Figure 3.

As seen in that figure, the inputs are the original and segmented versions of the cervical smear images. In the first stage of the proposed technique, features relevant to the colour, shape, or texture of the input smeared images are extracted. Following this, in the second stage, the proposed QH (i.e., the combination of QPSO and Fuzzy *k*-NN) algorithm is used for feature selection and subsequent cell classification. The output of the system provides an inference about the predicted class from data testing of cervical smear images.

**Figure 4:**
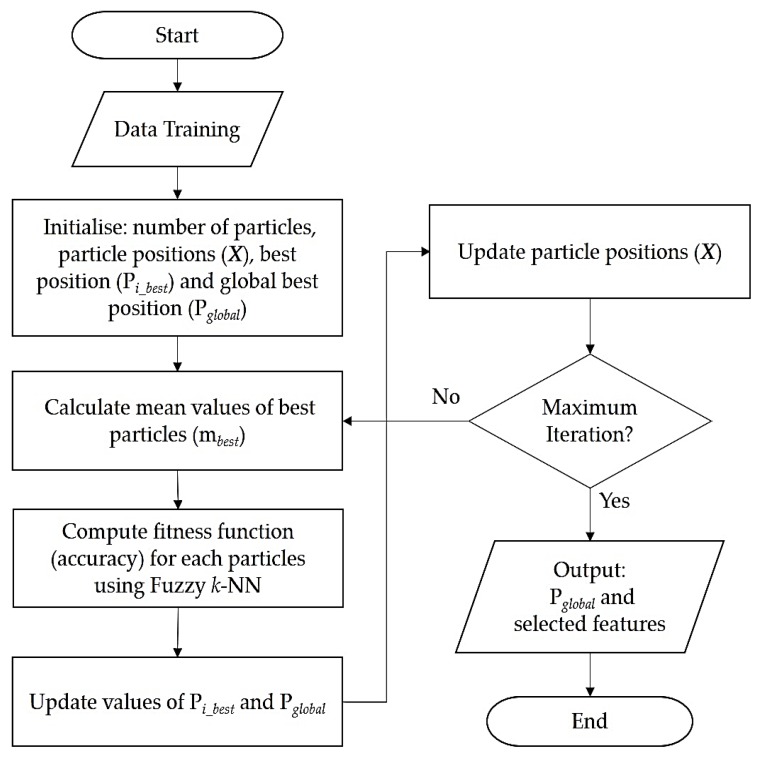
Flowchart depicting process of feature selection using the proposed Q-Fuzzy approach.

The flowchart in Figure 4 further highlights the steps of our proposed feature selection and classification using proposed the QH or Q-Fuzzy (i.e., QPSO blended with Fuzzy *k*-NN) approach.

To ensure effective cell classification, the feature extraction stage is designed to target seventeen (17) features related to the geometry, colour, and texture of the input CS images. These features have been widely cited in previous studies, notably [3,11,13]. However, for integrality, we further define these features in Equations (10)–(26).

- Area of nucleus, *A_n_*
  *A_n_* = number of pixels in the nucleus region (10)
- Major axis of nucleus, *L_n_*
  *L_n_* = the length of the major axis of an ellipse that completely encloses the nucleus region. (11)
- Minor axis of nucleus, *D_n_*
  *D_n_* = the length of the minor axis of an ellipse that completely encloses the nucleus region. (12)
- Aspect ratio of nucleus, $R_{n}$
  (13) $$R_{n}=\frac{W_{n}}{H_{n}},$$
  where *Wn* is the width of the nucleus and *Hn* is the height of the nucleus region.
- Perimeter of Nucleus, *P_n_*
  *P_n_* = the perimeter of the nucleus region, (14)
- Roundness of nucleus, *N_circle_*
  (15) $$N_{circle}=\frac{\pi }{4}.{L_{n}}^{2}\to N_{roundness}=\frac{A_{n}}{N_{circle}}.$$
- Maxima of nucleus, *Max_n_*
  *Max_n_* = number of local maximum value from eight pixels in the neighbourhood of the nucleus region. (16)
- Minima of nucleus, *Min_n_*
  *Min_n_* = number of local minimum value from eight pixels in the neighbourhood of the nucleus region. (17)
- Homogenity of nucleus, $H_{n}$
  (18) $$H_{n}=\overset{m}{\underset{i=1}{\sum }}\overset{m}{\underset{i=1}{\sum }}\frac{p\left(i,j\right)}{1+\left\lceil i-j\right\rceil },$$
  where *p*(*i*, *j*) is the probability pixel pairs in the nucleus region calculated by gray level co-occurrence matrix and *m* is the number of grey level in image.
- Brightness of nucleus, *B_n_*
  *B_n_* = the average pixel intensity of nucleus region. (19)
- Maxima of cytoplasm, *Max_c_*
  *Max_c_* = number of local maximum value from eight (8) pixels in the neighbourhood of the cytoplasm region. (20)
- Minima of cytoplasm, *Min_c_*
  *Min_c_* = number of local minimum value from eight (8) pixels in the neighbourhood of the cytoplasm region. (21)
- Brightness of cytoplasm, *Bc*
  *Bc* = the average pixel intensity of cytoplasm region. (22)
- Area of entire cell, *A_cell_*
  *A_cell_* = number of pixels in the cell region. (23)
- Compactness of the entire cell, $C_{cell}$
  (24) $$C_{cell}=\frac{{P^{2}}_{cell}}{A_{cell}}.$$
- Ratio of nucleus and cell, $R_{cell}$
  (25) $$R_{cell}=\frac{A_{nu}}{A_{cy}},$$
  where *Acell* is area of the *cell* region
-  
  *LBP_cell_* = the local binary pattern of cell region. (26)

To extract textural features from the CS images, we use Local Binary Pattern Histogram Fourier (LBP-HF) protocol, wherein we start by applying operator LBP to obtain the pattern of data and create its histogram using Uniform LBP approach. Second, the LBP-HF feature is computed by applying Discrete Fourier Transform (DFT) from *n* histogram Uniform LBPs. Finally, the feature vector of LBP-HF is obtained by combining histogram values of all zeros, all ones, non-uniform, and Fourier spectrum values.

Based on the LBP-HF and relevant texture features in Equations (10)–(26), we obtain a texture feature vector of 38 entries, while the total feature vector has 54 entries. As mentioned earlier, further details about these features can be found in [3,10,13].

As outlined (in the red short-dashed rectangle) in Figure 3, the feature selection stage of our proposed QHT technique comprises of two units that utilise the quantum-behaved PSO technique and the Fuzzy *k*-NN algorithm so that both feature selection and classification accuracy in smeared cervical images are enhanced. 

We further clarify that (in Figure 4) the fuzzy *k*-NN is applied prior to computing the fitness function, i.e., using the F1 score (defined later in Equation (29)). In other words, the QPSO approach is used to find the variation of subset features by generating particles that are each evaluated by calculating fitness values from the accuracy of classification result. Following this, the Fuzzy *k*-NN method is applied to classify the categories of smear images. Based on their fitness values, the particles with best fitness values are assigned as local best position and global best position. The particle that is assigned as global best position represents the best subset features.

In this study, we shall use Fuzzy *k*-NN to enhance the accuracy of our classification of cells in smeared cervical images, and the best position *P_i_best_* of particle *i* is determined using Equation (18) [18].

## 4. Experiments on Smeared Cervical Images Using Q-Fuzzy Approach

Using the framework outlined in Figure 3 and Figure 4, which were discussed in latter parts of the preceding section, in this section, we present an experimental validation regarding the utility of our proposed QH approach for feature selection in cervical cell classification.

### 4.1. Description of Dataset

For our experimental validation, the unit cell microscopic cervical smear (CS) images from the Herlev dataset [24] will be used as input dataset. This dataset consists of 917 CS images that were collated by cytology experts using a microscope connected to a digital camera. Each image was taken with a resolution of 0.201 μm/pixel [10,11,12].

**Figure 5:**
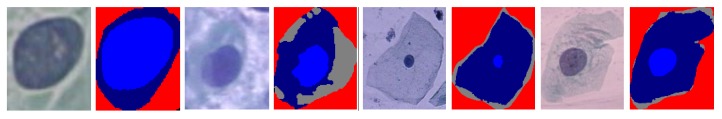
Sample cervical smear images and manual segmentation (ground truth) images [2,11,12,13,15,16,17].

After capturing the images, cytology experts were tasked with manually classifying the CS images into the seven classes discussed in Section 2. Each cell is assessed by two experts while a medical doctor was asked to further examine the samples that are deemed hard to deal with. All the images in the dataset are segmented into the cytoplasm, nucleus, and background regions using CHAMP software [12]. Results of the segmentation were further examined by cytology experts to ensure accuracy. A few sample images from the Herlev dataset (with their respective ground truth images) are shown in Figure 5. Furthermore, Table 4 presents the description of the Herlev smeared cervical dataset as well as their distribution and categories.

### 4.2. Evaluation Method

Many approaches are utilised to evaluate the performance of classification algorithms. Here, we use the Precision, Recall, and F1 Score analysis (or PRS analysis). Precision is used to ascertain the accuracy of classification using the number of correctly classified positive examples divided by the number of examples labelled by the system as positive (Equation (27)). Recall is the number of correctly classified positive examples divided by the number of positive examples in the data (Equation (28)), and F1 Score is a combination of Precision and Recall [30,31] as defined in Equation (29).

(27) $$\mathrm{Precision}\left(P\right)=\frac{tp}{tp+fp}$$

(28) $$\mathrm{Recall}\left(R\right)=\frac{tp}{tp+fn}$$

(29) $$F1\;\mathrm{score}=2.\frac{P\times R}{P+R}$$

where *tp* is true positive, *fp* is false positive, and *fn* is false negative.

The *r*-fold cross validation method is employed for validating the experimental results, which considering the size of our data, 5-fold cross-validation is used.

To establish the relationship between the different feature classification approaches, we employ Cohen’s kappa statistical measure, which is widely used to quantify agreement between two raters (i.e., mechanism to assess or observe a variable or system), that each classify N items into C mutually exclusive categories [32] as defined in Equation (30).

(30) $$\kappa =\frac{p_{o}-p_{e}}{1-p_{e}}$$

where $p_{o}$ is the relative observed agreement between raters (akin to accuracy) and $p_{e}$ is the hypothetical probability of chance agreement. If raters are in complete agreement κ=1, whereas when there is no agreement among the raters other than what would be expected by chance (i.e., as given by $p_{e}$), then κ≤0.

### 4.3. Experimental Result for Cervical Smear Image Classification

Earlier in Section 3, the mechanism via which the best out of an assemblage of 17 features with size of feature vector of 54 entries for the CS images were pruned down to seven features with a feature vector of 32 entries. 

The seven best features utilised for the remainder of our performance assessment are area of nucleus (i.e., Equation (10), roundness of nucleus i.e., Equation (15)), brightness of nucleus (i.e., Equation (19)), brightness of cytoplasm (i.e., Equation (22)), area of entire cell (i.e., Equation (23)), ratio of nucleus and cytoplasm (i.e., Equation (25)), and local binary pattern with histogram Fourier (LBP-HF) (i.e., Equation (26)). Furthermore, for the LBP-HF, 25 of 38 feature vectors were selected. Meanwhile, in the Fuzzy *k*-NN unit of the proposed Q-Fuzzy approach (Figure 3), we computed the accuracy for varying values of *k* (i.e., the number of nearest neighbours as outlined earlier in Section 2 and discussed in standard literature including [22,23]) starting with *k* = 2, which implies that at least 2 nearest neighbours are considered in determining the prediction result. In addition, it was observed that beyond *k* = 4 the accuracy of prediction results decreased, and so the upper bound was limited at *k* = 7.

We clarify here that our experiments are mainly focused on establishing the impact of the feature selection on the classification accuracy and how well our proposed QH approach (which combines the QPSO algorithm with the Fuzzy *k*-NN—or simply the Q-Fuzzy approach) contributes toward improving the performance of classification results, which is deemed crucial for cervical cancer detection.

Consequently, we considered three experimental scenarios whereby, in the first two experiments, we compared the classification results obtainable with and without prior feature selection. This encompasses comparisons between our proposed Q-Fuzzy approach and other Fuzzy *k*-NN hybrid methods including one that blends the standard PSO with the Fuzzy *k*-NN technique (which we refer to as the P-Fuzzy approach) alongside the All-Features approach, i.e., classification without prior feature selection imposing the use of all the image features. Based on this setup, in the first experimental scenario, we present an assessment of the classification results for the All-features, P-Fuzzy, and Q-Fuzzy approaches.

For a more objective assessment, we maintained the same parameter values of PSO and QPSO as well as the number of particles and number of iterations (maintained as 20 and 200, respectively) while varying the number of nearest neighbours, *k*. The outcomes (in Table 5) show that (for all values of *k*) applying prior feature selection (i.e., in both the P-Fuzzy or Q-Fuzzy approaches) improved the classification results in comparison to instances using all features (i.e., the All-Features approach). The highest performance obtained without feature selection (i.e., using all features) is realised at *k* = 5, whereas the best performance for the P-Fuzzy and Q-Fuzzy approaches was obtained at *k* = 4.

Furthermore, using the same approaches (i.e., All-features, P-Fuzzy, and Q-Fuzzy), in the second experimental scenario, we assessed the impact of prior feature selection on the classification accuracy for all the seven cervical cell categories. Our results, in Table 6, indicate that our proposed QH feature selection approach that blends the QPSO algorithm with the Fuzzy *k*-NN (i.e., the Q-Fuzzy approach) yielded better classification accuracy than both the All-features and P-Fuzzy approaches in terms of outcomes of the PRS analysis. Further investigation of the outcomes indicates an improvement in the classification accuracy for all cell categories when prior feature selection (P-Fuzzy or Q-Fuzzy) was utilised. Moreover, our proposed Q-Fuzzy approach outperformed the P-Fuzzy hybrid approach for all cervical cell categories (except the Normal Columnar category) with its best classification results in the Normal superficial cell category.

To further establish the utility of the proposed Q-Fuzzy QH approach, in the third experimental scenario, we compared it (i.e., the proposed Q-Fuzzy approach) with other hybrid QH approaches formed by blending the QPSO algorithm with other supervised learning methods, namely, the Naïve Bayes (NB) and the support vector machines (SVM). Additionally, we examined the performance of the proposed approach alongside other feature selection methods, such as the filtering approach i.e., Relief [31,32] (by selecting the 25 top-ranked features from a feature vector of 54 entries (i.e., based on the Relief score), the sequential feature selection (SFS) method, which has an in-built capability to add or remove features sequentially [33,34] and the random forest (RF) method, which is an ensemble learning based technique that is widely employed in classification, regression, etc. [31].

To clearly assess the potential impacts of integrating ‘quantumness’ into cell classification of cervical smear images for the purpose of cervical cancer detection, our discussion of the results in Table 7 will be predicated in two directions.

First, we analyse the results in terms of the resulting HIS models that are realised by blending the classifiers (in the extreme left of Table 7) with the feature selection methods (Naïve Bayes (NB) support vector machines (SVM) and random forest (RF)) that produce the NB, RF, SVM, and Fuzzy *k*-NN (F*k*NN) HIS models. To simplify our discussion, we further divided the models into two categories. The first category consists of non-quantum HIS models wherein neither the classifier nor the feature selection units has any ‘quantumness’ in it. The second category comprises of quantum HIS techniques (or simply quantum hybrid techniques) that are realised by blending the QPSO feature selection method with each of the four classifiers (NB, SVM, RF, and F*k*NN). The resulting NB, SVM, RF, and F*k*NN QHTs are shown highlighted in Table 7. Notably, we clarify that the F*k*NN QHT (whose results are shaded in darker background) is also our proposed Q-Fuzzy approach that blends the QPSO into the Fuzzy *k*-NN classifier method. 

Our analysis in terms of the HIS models pitches the non-quantum models against the QH techniques for each classifier method. As reported in Table 7, the QHT technique of each classification method (i.e., QPSO blended with NB, QPSO blended with SVM, QPSO blended with RF and QPSO blended with F*k*NN) present between marginal to modest improvements across all the parameters (i.e., Precision, Recall, F1 score, and Cohen’s Kappa measure) used to assess the classification accuracy than most of the non-quantum HIS models for that same classifier. Similarly, the results obtained when All-Features (i.e., without prior feature selection) methods is used are improved along all parameters in comparison with using the QHT of each corresponding classification method.

To focus the assessment in terms of our proposed Q-Fuzzy approach, the second perspective of our discussion of the results in Table 7 is confined to comparisons between the four QHTs. The results (shaded area) show that, with the exception of the RF-based QHT, our proposed Q-Fuzzy approach (or F*k*NN-based QHT) performs better than the NB and SVM-based QHTs in terms of all four parameters that are reported (in Table 7). Even though these improvements are modest (ranging between 1 to 9 percent for Precision, Recall and F1 score and 2 to 11 percent for the Cohen’s Kappa statistical measure) their impact in terms of better detection of cancerous cells in cervical smear images are significant. Moreover, as reported earlier in Table 6, our proposed Q-Fuzzy approach outperformed its closest competition (the P-Fuzzy technique or PSO-based non-quantum HIS) in terms of accuracy of classifying most of the cell categories in the cervical smear images. Similarly, as reported in Table 5, the proposed Q-Fuzzy approach presented better classification accuracy in terms of abnormal cell categories than the P-Fuzzy method.

In concluding, we reiterate that all along our objective has been to explore the impact of integrating ‘quantumness’ into HIS models. As reported earlier in this section, the quantum hybridised QHT models enhanced the classification accuracy across most of classifier methods (i.e., NB, SVM and Fuzzy *k*-NN) that were evaluated. Furthermore, as noted in earlier Section 2, the improvements recorded via the proposed Q-Fuzzy approach could be attributed to the manner that the intuitionistic rationality of the Fuzzy *k*-NN complements the adaptive search capability of the quantum-behaved QPSO algorithm. Consequently, the foregoing narrative enunciates potential applications for quantum machine learning algorithms in image-based approaches to disease diagnosis and treatment.

## 5. Concluding Remarks

Accuracy is an important aspect of cell detection and delineation, especially in many image-based applications for disease diagnosis. To enhance the accuracy of classification of cells in smeared cervical images, our study proposes a hybrid feature selection technique that combines the potency of the quantum-behaved particle swarm optimisation (QPSO) algorithm with the versatility of the Fuzzy *k*-nearest neighbours (Fuzzy *k*-NN) algorithm (i.e., the proposed quantum hybrid (QH) or Q-Fuzzy technique). 

From an initial multitude of 17 features that describe the geometry, colour, and texture of the cervical images, the QPSO stage of our proposed technique is used to select the best subset features (i.e., global best particles) that represent a pruned-down collection of seven features.

Using the Herlev dataset [24]) consisting of almost 1000 images, our verification of the cogency of the proposed QH approach in cervical cancer detection was predicated upon its use in three experimental scenarios. The purpose of the first two scenarios is to establish the impact of prior feature selection on classification accuracy. In these experiments, we compared the All-Features approach (i.e., when no prior feature selection is used in the classification) alongside HIS models including the P-Fuzzy approach (which is a hybrid approach that combines the standard PSO algorithm with the Fuzzy *k*-NN technique) on one side and alongside our proposed Q-Fuzzy approach that blends the quantum-behaved PSO algorithm with the Fuzzy *k*-NN technique. Outcomes from these tests confirm that integrating ‘quantumness’ into the feature selection units led to enhanced classification accuracy. Moreover, the efficacy of our proposed Q-Fuzzy approach manifests in the manner that it rivals the P-Fuzzy approach in terms of classification accuracy for six out of the seven cervical cell categories. In the third experimental scenario, we established the synergy between the quantum-behaved PSO and the Fuzzy *k*-NN technique that form our proposed Q-Fuzzy approach by comparing its classification accuracy alongside that from a trio of hybrid QH approaches that each blends the QPSO with other classification methods, namely the Naïve Bayes (NB) networks, the random forest (RF) ensemble method and the support vector machine (SVM). Outcomes establish that the adaptive search capability of the quantum-behaved QPSO algorithm is best complemented by the intuitionistic rationality inherent to the Fuzzy *k*-NN technique. Overall, our proposed QPSO blended Fuzzy *k*-NN QH approach (i.e., Q-Fuzzy approach) outperformed the other approaches that QPSO blended with the NB and SVM with modest average increases ranging from 2 to 11 percent in terms of the classification parameters that were evaluated.

Buoyed by the foregoing outcomes, in ongoing work, additional ‘quantumness’ will be exploited and integrated into the QPSO (as well as in QH techniques generally) so that the accuracy and performance of the feature selection could be further enhanced. These efforts would improve the effectiveness of image-based approaches used in cervical cancer diagnosis and treatment. Later, we hope to exploit these strategies (and others in [35]) in image-based detection of other carcinogens.

## Figures and Tables

**Table 1:** Classification of Pap smear single image cell types.

1	Normal cells	Superficial squamous
		Intermediate squamous
		Columnar
2	Abnormal cells	Mild dysplasia
		Moderate dysplasia
		Severe dysplasia
		Carcinoma in situ

**Table 2:** Pseudocode for executing the QPSO algorithm [18,19].

Initialise the current positions and the *P_i_best_* positions of all the particles
Do
Calculate *m_best_* in Equation (2)
Select a suitable value for *α*
For particles *i* = 1 to *S*
1. Calculate the fitness value of particle *i* according to classification accuracy
2. Update *P_i_best_* and *Pglobal* in Equation (7)
3. For dimension 1 to *d*
*φ* = rand (0,1)
*u* = rand (0,1)
If *s* = rand (0,1) ≥ 0.5
update particle positions in Equation (4)
else
update particle positions in Equation (3)
Until terminal condition is satisfied.

**Table 3:** Pseudocode for executing the Fuzzy *k*-NN algorithm [22,23].

Normalise the data
Find the *k*-nearest neighbours
Calculate the membership value *u*(*x*, $c_{i}$) in Equation (9)
Select the maximum value of *c* from *u*(*x*, $c_{i}$)
Assign class *c* to the data

**Table 4:** Classification of Pap smear single image cell types in the Herlev dataset [24].

Cell	Class Name	Cell Count	Sub-Total
Normal	Normal superficial squamous	74	242
	Normal intermediate squamous	70	
	Normal columnar	98	
Abnormal	Carcinoma in situ	150	675
	Light dysplastic	182	
	Moderate dysplastic	146	
	Severe dysplastic	197	
	**Total**	**917**	**917**

**Table 5:** Comparison of classification results for approaches with (i.e., P-Fuzzy and Q-Fuzzy) and those without prior feature selection (i.e., All-features).

*k*	All-Features				P-Fuzzy				Q-Fuzzy			
	Precision	Recall	F1 Score	κ	Precision	Recall	F1 Score	κ	Precision	Recall	F1 Score	κ
2	0.67	0.74	0.70	0.66	0.73	0.80	0.76	0.72	0.73	0.79	0.76	0.71
3	0.68	0.69	0.68	0.64	0.76	0.77	0.76	0.72	0.77	0.77	0.77	0.73
4	0.74	0.74	0.74	0.70	**0.83**	**0.84**	**0.83**	**0.81**	**0.85**	**0.86**	**0.85**	**0.83**
5	**0.76**	**0.76**	**0.76**	**0.72**	0.80	0.81	0.80	0.76	0.80	0.81	0.80	0.76
6	0.73	0.73	0.73	0.68	0.74	0.76	0.75	0.70	0.74	0.75	0.74	0.69
7	0.69	0.70	0.69	0.64	0.71	0.72	0.71	0.67	0.73	0.74	0.73	0.69

**Table 6:** Comparison of classification results with and without prior feature selection using the all-features, P-Fuzzy, and proposed Q-Fuzzy approaches.

Cell Category	All Features (*k* = 5)			P-Fuzzy (*k* = 4)			Q-Fuzzy (*k* = 4)		
	Precision	Recall	F1 Score	Precision	Recall	F1 Score	Precision	Recall	F1 Score
Normal superficial	0.83	0.86	0.84	0.95	0.91	0.93	0.95	0.95	0.95
Normal intermediate	0.82	0.74	0.78	0.89	0.84	0.86	0.89	0.89	0.89
Normal columnar	0.63	0.67	0.65	0.65	0.72	0.68	0.61	0.74	0.67
Carcinoma in situ	0.74	0.83	0.78	0.81	0.87	0.84	0.84	0.90	0.87
Light dysplastic	0.83	0.78	0.81	0.88	0.94	0.91	0.89	0.97	0.93
Moderate dysplastic	0.69	0.85	0.76	0.83	0.96	0.89	0.89	0.96	0.93
Severe dysplastic	0.79	0.62	0.70	0.86	0.65	0.74	0.88	0.61	0.72

**Table 7:** Comparison of classification outcomes between hybrid QH approaches that combine QPSO with Naïve Bayes, Support Vector Machines (SVM), Random Forest (RF), and Fuzzy *k*-NN.

Classifier Methods		All Features	Feature Selection Methods			
			Non-Quantum HIS			QHT
			Relief	SFS	PSO	QPSO
**Naïve Bayes** **(NB)**	Precision	0.72	0.75	0.76	0.73	0.76
	Recall	0.72	0.76	0.77	0.74	0.77
	F1 score	0.72	0.75	0.76	0.73	0.76
	κ	0.67	0.71	0.72	0.68	0.72
**SVM**	Precision	0.79	0.83	0.85	0.84	0.84
	Recall	0.79	0.83	0.86	0.84	0.85
	F1 score	0.79	0.82	0.84	0.84	0.84
	κ	0.74	0.80	0.82	0.81	0.81
**Random Forest** **(RF)**	Precision	0.79	0.83	0.85	0.84	0.85
	Recall	0.80	0.83	0.86	0.84	0.86
	F1 score	0.79	0.82	0.85	0.84	0.85
	κ	0.74	0.80	0.83	0.81	0.82
**Fuzzy *k*-NN** **(*k* = 4 and *k* = 5)**	Precision	0.76	0.74	0.80	0.83	0.85
	Recall	0.76	0.79	0.85	0.84	0.86
	F1 score	0.76	0.76	0.83	0.83	0.85
	κ	0.72	0.72	0.80	0.81	0.83
